# Strain-specific differences in pili formation and the interaction of *Corynebacterium diphtheriae *with host cells

**DOI:** 10.1186/1471-2180-10-257

**Published:** 2010-10-13

**Authors:** Lisa Ott, Martina Höller, Johannes Rheinlaender, Tilman E Schäffer, Michael Hensel, Andreas Burkovski

**Affiliations:** 1 Lehrstuhl für Mikrobiologie, Friedrich-Alexander-Universität Erlangen-Nürnberg, Nürnberg, Germany; 2 Lehrstuhl für Angewandte Physik, Friedrich-Alexander-Universität Erlangen-Nürnberg, Nürnberg, Germany; 3 Mikrobiologisches Institut des Universitätsklinikums Erlangen, Erlangen, Germany; 4 Abteilung Mikrobiologie, Universität Osnabrück, Osnabrück, Germany

## Abstract

**Background:**

*Corynebacterium diphtheriae*, the causative agent of diphtheria, is well-investigated in respect to toxin production, while little is known about *C. diphtheriae *factors crucial for colonization of the host. In this study, we investigated strain-specific differences in adhesion, invasion and intracellular survival and analyzed formation of pili in different isolates.

**Results:**

Adhesion of different *C. diphtheriae *strains to epithelial cells and invasion of these cells are not strictly coupled processes. Using ultrastructure analyses by atomic force microscopy, significant differences in macromolecular surface structures were found between the investigated *C. diphtheriae *strains in respect to number and length of pili. Interestingly, adhesion and pili formation are not coupled processes and also no correlation between invasion and pili formation was found. Using RNA hybridization and Western blotting experiments, strain-specific pili expression patterns were observed. None of the studied *C. diphtheriae *strains had a dramatic detrimental effect on host cell viability as indicated by measurements of transepithelial resistance of Detroit 562 cell monolayers and fluorescence microscopy, leading to the assumption that *C. diphtheriae *strains might use epithelial cells as an environmental niche supplying protection against antibodies and macrophages.

**Conclusions:**

The results obtained suggest that it is necessary to investigate various isolates on a molecular level to understand and to predict the colonization process of different *C. diphtheriae *strains.

## Background

*Corynebacterium diphtheriae *is the causative agent of diphtheria, a toxaemic localized infection of the respiratory tract. While this disease is well-controlled by vaccination against the diphtheria toxin in e. g. Western Europe [1-3], it is still a severe health problem in less developed countries. Furthermore, *C. diphtheriae *is not only the aetiological agent of diphtheria, but can cause other infections as well. Non-toxigenic strains have been increasingly documented [4-6] and found to be the cause of invasive diseases such as endocarditis, bacteraemia, pneumonia, osteomyelitis, spleen abscesses, and septic arthritis [7,8]. As indicated by these systemic infections, *C. diphtheriae *is not only able to attach to host epithelial cells of larynx and pharynx, but must be able to gain access to deeper tissues and to persist inside tissues or cells.

A possible clue for the background of persistence of *C. diphtheriae *came from investigations of adherence and invasion of toxigenic and non-toxigenic strains by different groups. Using a combination of gentamicin protection assays and thin-section electron microscopy, Hirata and co-workers [9] showed that toxigenic *C. diphtheriae *are not only able to adhere to laryngeal HEp-2 cells, but also enter these cells and survive after internalization. Similar observations were made for non-toxigenic strains [10] showing that also pharyngeal Detroit 562 cells can be invaded by *C. diphtheriae *and that viable intracellular bacteria can be detected up to 48 h after infection.

While host cell receptors and invasion-associated proteins of the pathogen are still unknown, bacterial adhesion factors have been recently at least partially characterized on the molecular level. *C. diphtheriae *strain NCTC13129 is able to assemble three distinct types of pili on its surface [11,12]. Mutant analyses showed that the SpaA-type pilus is sufficient for adhesion of this strain to pharynx cells, shaft proteins are not crucial for pathogen-host interaction, and adherence to pharyngeal cells is greatly diminished when minor pili proteins SpaB and SpaC are lacking [13]. The results obtained in other studies indicated the existence of additional proteins besides pili subunits involved in adhesion to larynx, pharynx, and lung epithelial cells, since a total loss of attachment to pharyngeal cells due to mutagenesis of pili- and sortase-encoding genes could not be observed and attachment to lung or larynx cells was less affected by the mutations. This is in line with a number of studies suggesting the multifactorial mechanism of adhesion (reviewed in [14]). Furthermore, Hirata and co-workers [7,15] described three distinct patterns of adherence to HEp-2 cells, an aggregative, a localized, and a diffuse form, an observation that hints also to the existence of several adhesion factors and different receptors on the host cell surface. The involvement of different *C. diphtheriae *proteins to adherence to distinct cell types is further supported by work on adhesion to human erythrocytes, showing that non-fimbrial surface proteins 67p and 72p, which were up to now only characterized by their apparent mass, are involved in this process [16]. Interestingly, besides strain-specific differences in adherences (see references cited above), also growth-dependent effects were observed. In a study using two toxigenic *C. diphtheriae *strains and erythrocytes as well as HEp-2 cells, de Oliveira Moreira and co-workers [17] showed an effect of iron supply on hemagglutination and lectin binding properties of the microorganisms.

In this study, we present a characterization of different non-toxigenic *C. diphtheriae *and a toxin-producing strain with respect to adhesion to and internalization into epithelial cells. Analyses reveal significant strain-specific differences in host colonization and macromolecular surface structures of the studied strains, while neither of the strains evoked rapid cell damage under the conditions tested.

## Results

### Adhesion of *C. diphtheriae *to epithelial cells, invasion of host cells and intracellular survival

**Figure 1:**
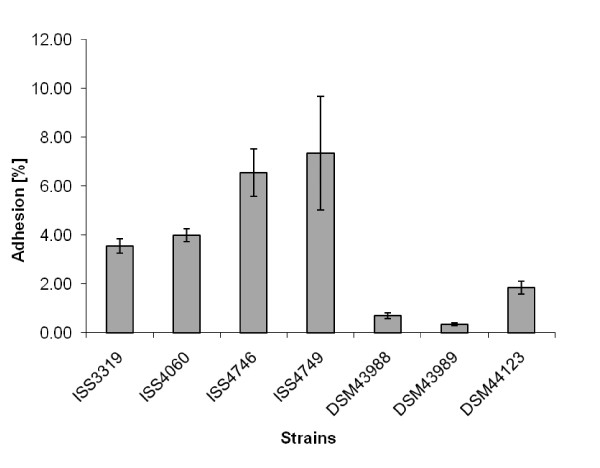
**Adhesion of *C. diphtheriae *strains to D562 cell layers**. D562 cells were infected with different *C. diphtheriae *strains. Besides DSM43989, which is *tox*^+^, the isolates are non-toxigenic. The cells were washed with PBS, detached with trypsin solution, lysed with Tween 20, and the number of colony forming units (cfus) was determined. Adhesion is expressed as percentage of the inoculum, showing means and standard deviations of ten independent measurements (biological replicates) with 3 samples each (technical replicates). All strains, except ISS4746 and ISS4749, show statistically significant differences in adhesion rates (students TTEST values below 0.04).

In this study, adhesion of six non-toxigenic strains and one toxin-producing *C. diphtheriae *to Detroit562 cells was analyzed (Fig. 1). In these experiments *tox*^+ ^strain DSM43989 showed the lowest adhesion rate with 0.34 ± 0.05%. While in general higher than the *tox*^+ ^strain, the non-toxigenic strains differed significantly in their adhesion rate, varying between 0.69 ± 0.12% for strain DSM43988 and 7.34 ± 2.33% for strain ISS4749 (Fig. 1).

**Figure 2:**
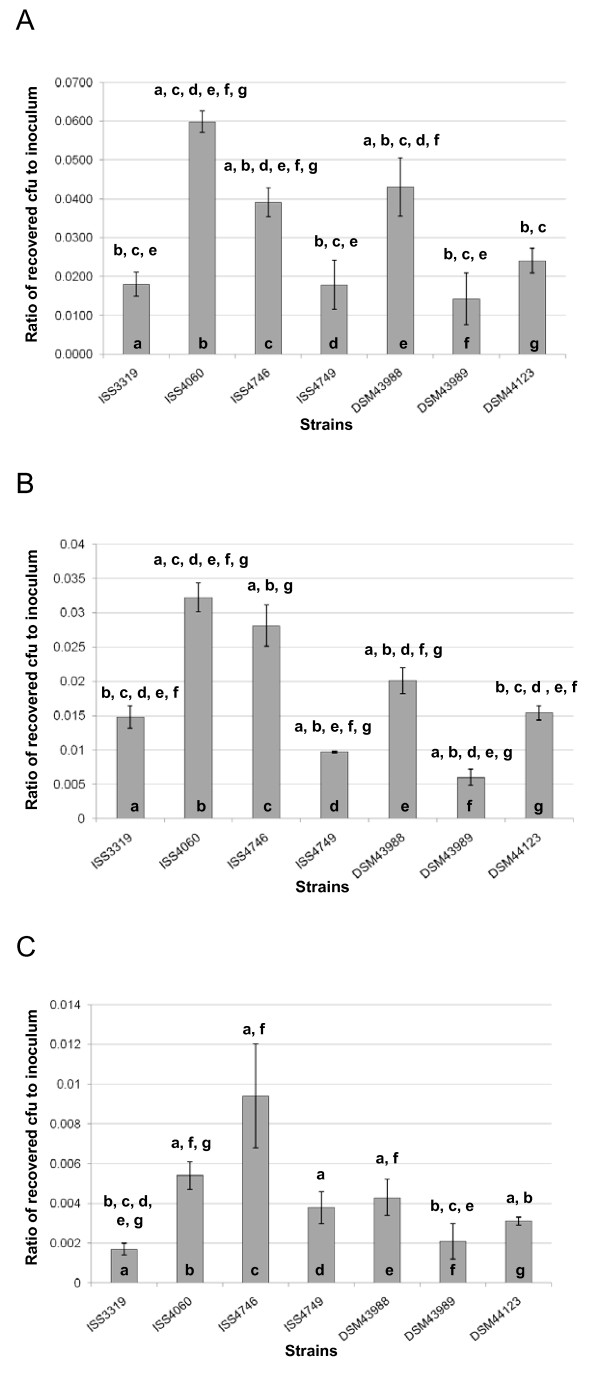
**Invasion of epithelial cells by *C. diphtheriae *strains**. D562 cells were infected with different *C. diphtheriae *strains (DSM43989 *tox*^+^, all others are non-toxigenic), washed, and incubated 2.0 (A), 8.5 (B) and 18.5 (C) hours with 100 μg ml^-1 ^gentamicin. Subsequently, cells were washed, detached with trypsin solution, lysed with Tween 20, and the number of intracellular cfus was determined. Invasion is shown as percentage of the inoculum internalized (means and standard deviations of three independent biological replicates with three samples each (technical replicates). Statistically relevant differences between the strains (based on students TTEST values below 0.05) are indicated by letters above columns.

Once attached to the surface of an epithelial cell, *C. diphtheriae *might invade the host cell and persist within the cell. In order to investigate this process for the different strains studied here, gentamicin protection assays were carried out. For this purpose, cells were incubated for 1.5 h with bacteria, gentamicin was added to kill remaining extracellular *C. diphtheriae *and survival of intracellular bacteria was analyzed after different times of incubation (Fig. 2). When invasion into D562 cells was analyzed for the six non-toxigenic strains and the toxigenic *C. diphtheriae *strain after 2 h, *tox*^+ ^strain DSM43989 showed the lowest internalization rate with 0.014 ± 0.007%. As in the adhesion assay, the non-toxigenic strains showed in general a higher rate compared to the toxin-producer strain and again rates differed significantly between the non-toxigenic strains, varying between 0.018 ± 0.006% for strain ISS4749 and 0.060 ± 0.027 for strain ISS4060 (Fig. 2A). The comparison of strains in respect to adhesion and internalization rates suggested that although a high adhesion seems to favour internalization, adhesion and invasion are not strictly coupled processes. Plating and counting of internalized cells after 8.5 and 18.5 h revealed decreasing numbers of colony forming units (Fig. 2B-C). Even after 18.5 h, no strain was completely eliminated from the cells and survival of bacteria ranged from 0.002 ± 0.001% of the inoculums for DSM43989 to 0.005 ± 0.001% for ISS4060.

**Figure 3:**
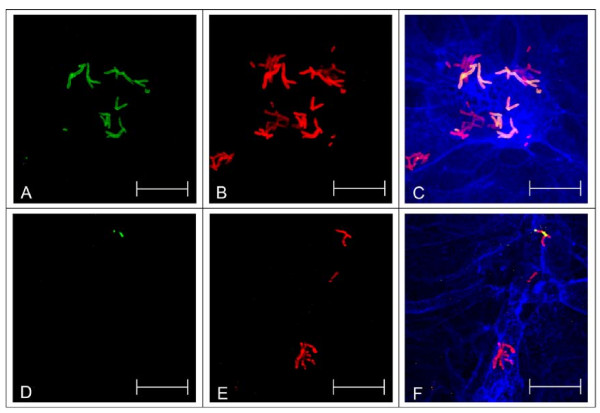
**Detection of intracellular *C. diphtheriae *in Detroit562 cells by immune-fluorescence microscopy**. D562 cells were seeded on coverslips 48 h prior to infection and infected with *C. diphtheriae *(DSM43989 *tox*^+^, all others are non-toxigenic) for 4 h with at a MOI of 200 as described earlier [26]. Antibodies directed against the surface proteome of *C. diphtheriae *were used as primary, Alexa Fluor 488 goat anti-rabbit IgGs and Alexa-Fluor 568 goat anti-rabbit IgGs as secondary antibodies (A, D: intact D562, B, E: permeabilized D562, C, F: overlay with blue F-actin stain Phalloidin-Alexa-Fluor 647, A-C: ISS3319, D-F: ISS4060. Green stain in panels A and D indicate extracellular bacteria. Dark red stain in panels B and E indicates internalized *C. diphtheriae*, while adherent bacteria appear in light red. In the overlay (C, F) extracellular *C. diphtheriae *appear orange, while internalized bacteria are stained dark red. Scale bars: 20 μm.

In addition to the gentamicin protection assay, which gives quantitative data, immune-fluorescence microscopy was applied as an independent method to investigate host cell interaction of *C. diphtheriae *strains. This method has the advantage of allowing direct visualization, although only on a qualitative level. Using an antiserum directed against *C. diphtheriae *surface proteins and antibody staining before and after permeabilization of the host cell, internalized *C. diphtheriae *were detected (Fig. 3). Interestingly, V-shaped *C. diphtheriae *dimers within the cells were observed. These V-forms are the result of the *Corynebacterium*-specific snapping division and indicate growing bacteria. Together with a tendency towards formation of clusters of cells (Fig. 3C and 3F), this observation suggests that bacteria replicate within the host cells and growth and elimination described above (Fig. 2A-C) are parallel processes.

### Influence of *C. diphtheriae *on the transepithelial resistance of cell monolayers

**Figure 4:**
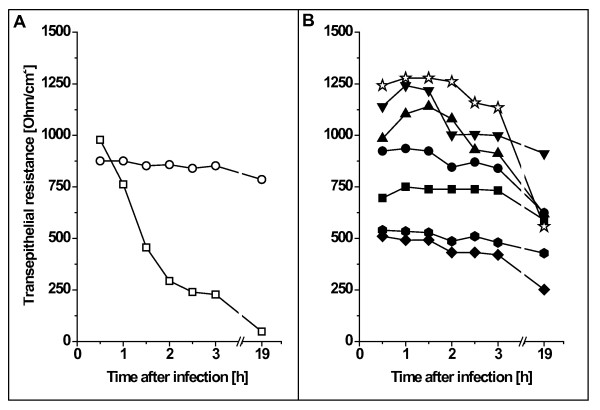
**Transepithelial resistance of polarized D562 monolayers grown on transwells**. (A) Control experiments of cells, which were incubated without bacteria (open circles) and *S. enterica *serovar Typhimurium (open squares). (B) Incubation with *C. diphtheriae *strains DSM43989 (*tox*^+^, open stars), ISS4749 (inverted closed triangles), ISS4746 (closed triangles), ISS4060 closed circles, ISS3319 (closed square), DSM43988 (closed hexagons), and DSM44123 (closed diamonds). Experiments were carried out independently at least thrice and typical results are shown.

Some pathogens, such as *Salmonella enteric *serovar Typhimurium (*S*. Typhimurium), can cause severe damage on cell membranes and due to the resulting loss of cell integrity, the transepithelial resistance of monolayers is dramatically reduced (for example see [18]). In this study, we used *S*. Typhimurium NCTC12023 as a positive control (Fig. 4A) and tested the influence of different *C. diphtheriae *strains on transepithelial resistance (Fig. 4B). Infection of Detroit562 monolayers with *S*. Typhimurium caused a dramatic break-down of transepithelial resistance within 1.5 h while all tested *C. diphtheriae *strains including *tox*^+ ^strain DSM43989 had no effect on transepithelial resistance within a time span of three hours. Since the incubation conditions used in this assay are similar to that applied for the adhesion and invasion assays, this observation indicates that a detrimental effect of toxin production can be excluded as a reason for the low adhesion and internalization rates of strain DSM43989.

Overnight incubation of D562 cells with *C. diphtheriae *was tested as well. In this case, the Dulbecco's modified Eagle's medium had to be exchanged after 3 h with fresh medium to remove not adhered bacteria in order to avoid that the pH of the medium dropped due to the bacterial metabolism leading to secondary detrimental effects. In contrast to short term incubation and to the non-toxigenic strains, long term measurement (Fig. 4B, overnight time point) of transepithelial resistance of cell monolayers infected with DSM43989 showed a significant effect, which might be caused by toxin production.

### Ultrastructural analysis of *C. diphtheriae *strains

**Figure 5:**
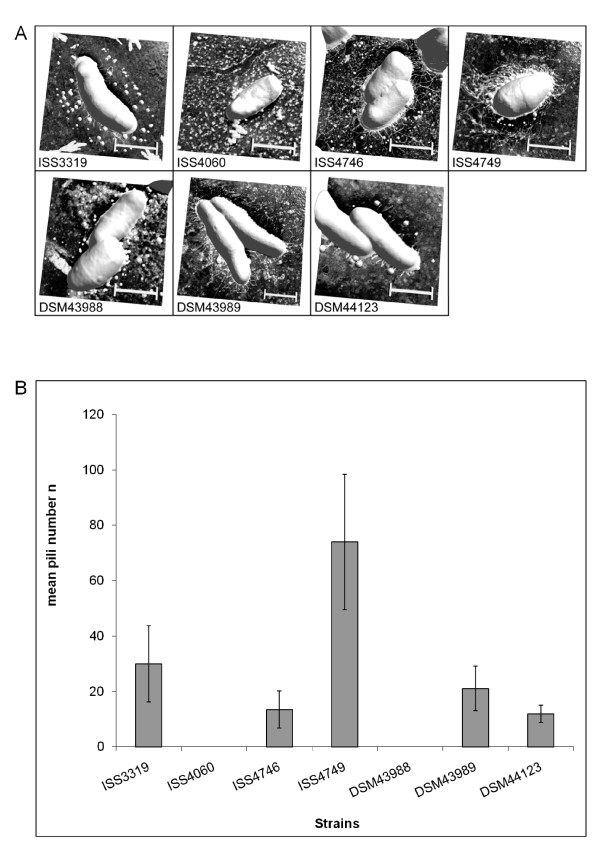
**Ultrastructural analysis of the cell surface of *C. diphtheriae *strains**. (A) Bacteria were fixed on glass slides by drying using compressed air. Atomic force microscopy was carried out under ambient laboratory conditions and operated in tapping mode. Scale bars: 500 nm. (B) AFM images were analyzed in respect to pili number per bacterium. For each strain pili of at least six bacteria were counted; error bars indicate deviations from mean values.

Since we suspected that the differences in adhesion might be the result of different surface structures, we started an ultrastructure analysis of selected *C. diphtheriae*. For this purpose, non-toxigenic strains as well as *tox*^+ ^strain DSM43989 were analyzed by atomic force microscopy (Fig. 5A). With this technique, which allows imaging surfaces topography at high resolution, significant different macromolecular surface structures were found between the different investigated *C. diphtheriae *strains. While for ISS4060 and DSM43988 pili were not detectable at all, ISS3319 and DSM44123 revealed short, spike-like pili, ISS4746, ISS4749 and DSM43989 showed long, hair-like protrusions (Fig. 5A). Also the number of pili (counted from at least six specimens of each strain) differed significantly (5B). Interestingly, adhesion and pili formation were not coupled, since ISS3319, which revealed spike-like pile and ISS4060, lacking these, showed comparable adhesion rates, while ISS4746 and ISS4749 had different numbers of long hair-like pili but showed identical adhesion rates. Also no correlation between invasion and pili formation was found. Since strain-specific differences in pili formation have not been observed before, the background for this phenomenon was investigated in more detail in subsequent experiments.

### Strain-specific expression of pili subunits

**Figure 6:**
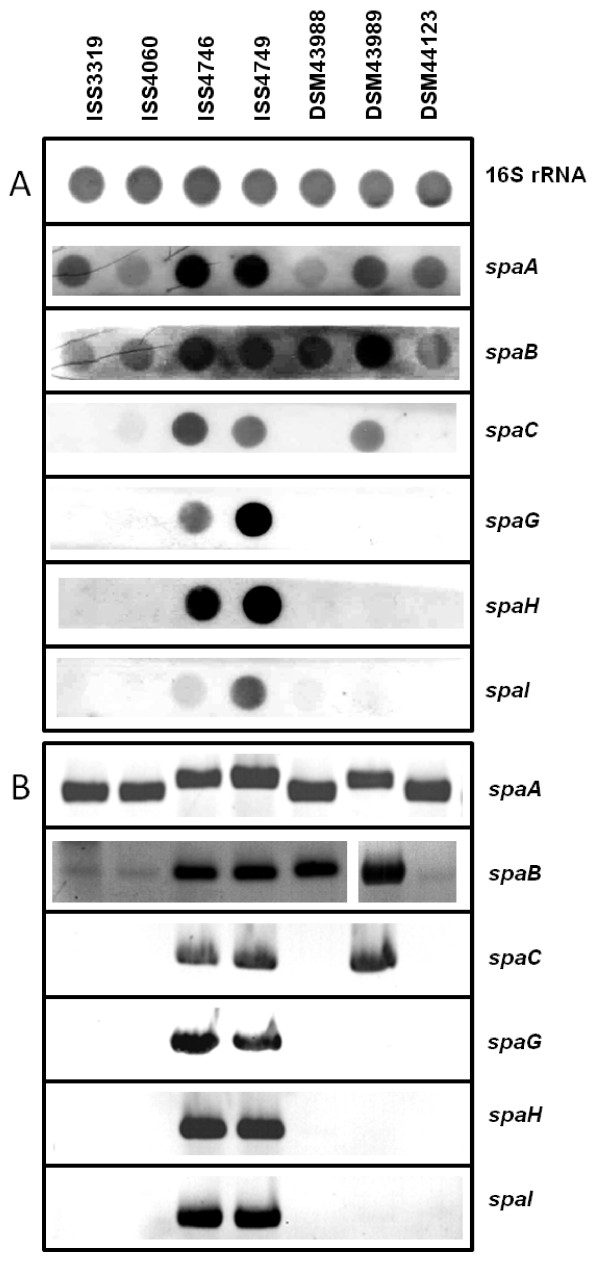
**Strain-specific distribution and expression of pili-encoding genes**. (A) Levels of *spa *gene transcripts in different *C. diphtheriae *strains. Total RNA was isolated from the indicated *C. diphtheriae *strains and hybridized with probes monitoring 16SrRNA for control as well as *spa *gene transcription. (B) PCR detection of *spa *genes. Chromosomal DNA of the indicated *C. diphtheriae *strains was used as template for PCR using specific oligonucleotide pairs for the *spa *genes indicated at the right side of the figure.

To analyze the molecular basis of strain-specific differences in pili formation, RNA hybridization experiments were carried out to study the mRNA levels of the *C. diphtheriae spa *genes. These genes are organized in three different clusters together with the corresponding sortase-encoding genes in the sequenced strain NCTC13129 [13,19]. The first cluster comprises the genes *spaA*, *spaB*, and *spaC*, which are most likely organized as an operon; the second cluster is formed by *spaD *and a putative *spaE-spaF *operon, and a third cluster comprises the *spaG*, *spaH*, and *spaI *gene, which are most likely independently transcribed. Strain-specific differences were detected, when probes for the detection of all genes of cluster I and III were applied in RNA hybridization experiments (Fig. 6A).

Strongest hybridization signals with *spaA*, *spaB*, and *spaC *probes were detected with RNA isolated from strains ISS4746 and ISS4749, slightly lower signal intensities were observed with strain DSM43989, while only faint signals were obtained for cluster I mRNA for the other investigated strains. Strong transcription of *spaG*, *spaH*, and *spaI *were again detected in strains ISS4746 and ISS4749, while other strains did not express cluster III genes deduced from RNA hybridization experiments. The data are in accordance with the AFM experiments presented in Fig. 5, which show formation of a high number of extended pili for strains ISS4746 and ISS4749, followed by DSM43989; however, hybridization signals may differ not only due to mRNA abundance, but also due to sequence alteration.

**Figure 7:**
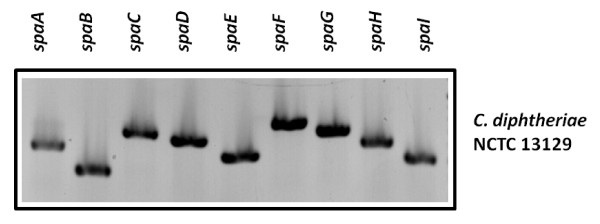
**PCR detection of *spa *genes in *C. diphtheriae *strain NCTC 13129**. Chromosomal DNA of *C. diphtheriae *strain NCTC 13129 was used as template for PCR using specific oligonucleotide pairs for the indicated *spa *genes. In all cases, DNA fragments of the expected size were amplified.

To elucidate whether the missing transcripts in various strains are the result of regulatory processes or have genetic reasons, PCR experiments were carried out, which showed that missing transcripts are correlated to lacking PCR products making regulatory effects unlikely (Fig. 6B). Furthermore, reproducible strain-specific differences in sizes of the PCR products were observed for *spaA *and in band intensities for *spaB *fragments, suggesting that also sequence deviations exist besides strain-specific differences in the *spa *gene repertoire. Interestingly, we were unable to generate any probes for genes of cluster II, comprising *spaD*, *spaE*, and *spaF*, when chromosomal DNA of the used ISS and DSM strains were used as template, while all genes could be amplified from NCTC13129 DNA (Fig. 7). Deduced from these PCR experiments, these genes seem to be absent in the investigated *C. diphtheriae *strains. As an additional approach, we tested expression of SpaD in the different strains by Western blot experiments. Cell extracts of strains ISS3319, ISS4040, ISS4746, ISS4749, DSM43988, DSM43989, and DSM44123 as well as purified SpaD protein as a positive control were separated by SDS-PAGE and subjected to Western blotting. SpaD-specific antiserum reacted exclusively with the SpaD control, while no signal was detectable in the investigated cell extracts (data not shown).

To address the hypothesis that pili expression patterns might change, when bacteria were in exposed to host cells, Green fluorescent protein (GFP) fluorescence of *C. diphtheriae *transformed with plasmids carrying *spa *gene upstream DNA and a promoter-less *gfp*uv gene was determined without and after 1.5 h of host cell contact. However, analysis of 80 to 140 bacteria for GFP fluorescence before and after host cell contact revealed no significant differences (data not shown).

## Discussion

In this study, different non-toxigenic *C. diphtheriae *and a toxin-producing strain were characterized in respect to adhesion to and invasion of epithelial cells. All strains were able to attach to host cells and immuno-fluorescence microscopy revealed internalization and growth of *C. diphtheriae *within epithelial cells. We could show that adhesion and invasion are not strictly coupled, indicating that different proteins and mechanisms play a role in these processes. Despite the fact that the number of internalized bacteria decreased over time for all investigated strains, a considerable number of bacteria survived prolonged internalization for more than 18 h. Furthermore, V-shaped division forms as well as formation of microcolonies were observed by fluorescence microscopy, suggesting that the epithelial cells might support growth of *C. diphtheriae*.

While proteins responsible for invasion and intracellular persistence are completely unknown for *C. diphtheriae*, for the sequenced strain NCTC13129 the influence of pili subunits on adhesion was characterized recently. It was shown that the minor pili subunits SpaB and SpaC are crucial for adhesion of strain NCTC13129 to epithelial cells [13], while pili length is influenced by the major pili subunits SpaA, SpaD, and SpaH, which form the shaft of the structure [11,12,19]. The strains investigated here showed significant variations in adhesion rates and compared to the *tox*^+ ^strain, for all studied non-toxigenic strains higher adhesion rates were observed; however, adhesion and pili formation were not strictly coupled processes. Strain-specific differences of appearance and numbers of pili-like structures on the surface of *C. diphtheriae *strains were shown by ultrastructural analyses *via *atomic force microscopy. Additionally, RNA hybridization and Western blotting experiments revealed distinct differences in the expression patterns of pili subunits for the investigated strains. To our knowledge, this is the first time that isolate-specific differences in pili formation were characterized. Mandlik and co-workers [13] showed that type III pili length of strain NCTC13129 depends on *spaH *expression and can be manipulated by deletion or overexpression of *spaH*. These results are supplemented here by showing that this is a phenomenon which occurs also as natural variation in different *C. diphtheriae *wild type isolates. Strains ISS4746 and ISS4749 showed the most extended pili structures, an observation which is correlated with high expression of *spaA *and *spaH *in these strains, while medium-length pili of DSM43989 are correlated with lack of *spaH *expression. As mentioned above, it was shown by mutant analyses of strain NCTC13129 that expression of *spaB *and *spaC *is crucial for adhesion to D562 cells [13]. Natural variations of the *spaB *and *spaC *expression patterns observed here indicate that this correlation is not as strict as suggested, since strain ISS4060 shows only low *spaB *and no *spaC *expression but a high adhesion rate, indicating that other factors are important for adhesion as well and expression of these might differ in various isolates.

The lack of any PCR product for *spaD*, *spaE*, and *spaF *and the absence of a SpaD signal in Western blotting experiments suggest that these genes are absent in the investigated strains. All pili-encoding genes of *C. diphtheriae *are located on pathogenicity islands [20,21]. Based on the genome sequence of strain NCTC13129, *C. diphtheriae *possesses 13 of these genomic islands [20,22] and pili cluster II is located on genomic island CDGI-2, which has a size of 17.5 kb and is located directly adjacent to 36.5 kb pathogenicity island CDGI-1, the *tox*^+ ^corynephage [20]. Data of PCR experiments (not shown) indicate that the pili-encoding genes located on CDGI-2 are missing in all investigated ISS and DSM strains and consequently the genetic repertoire of *C. diphtheriae *isolates is rather variable. This observation is in agreement with a recent genome survey of *C. diphtheriae *C7(-) and PW8 strains [23] indicating that 11 of the 13 putative pathogenicity islands of the sequenced reference strain NCTC13129 are absent in the C7(-) strain.

The importance of bacterial appendices and surface proteins for host cell contact were also shown recently for a non-fimbrial protein, DIP1281, previously annotated as invasion-associated protein. This protein is a virulence factor involved in cell surface organization, adhesion and internalization in epithelial cells. Corresponding mutant strains lack the ability to adhere to host cells or invade these [24].

## Conclusions

The results obtained in this study show that adhesion and invasion are not necessarily coupled processes. Adhesion rates are not strictly correlated with pili formation and in summary the pili repertoire of the investigated strains is highly variable. As shown by genome comparisons [23] it is necessary to investigate various isolates on a molecular level to understand and to predict the colonization process of different *C. diphtheriae *strains.

## Methods

### Bacterial strains and growth

Strains used in this study are listed in Table 1. *C. diphtheriae *strains were grown in Heart Infusion (HI) broth or on Columbia agar with sheep blood (Oxoid, Wesel, Germany) at 37°C. *S*. Typhimurium and *Escherichia coli *DH5αMCR were grown in Luria Broth (LB) [25] at 37°C. If appropriate, kanamycin was added (30 μg ml^-1 ^for *E. coli*; 50 μg ml^-1 ^for *C. diphtheriae*).

**Table 1:** Bacterial strains and eukaryotic cell lines used in this study

Strains	Description	Reference
***C. diphtheriae***		

DSM43988	non-toxigenic, isolated from throat culture	DSMZ, Braunschweig, Germany

DSM43989	*tox*^+^, unknown source	DSMZ, Braunschweig, Germany

DSM44123	non-toxigenic isolate, type-strain, unknown source	DSMZ, Braunschweig, Germany

ISS3319	*C. diphtheriae *var. *mitis*, non-toxigenic, isolated from patients affected by pharyngitis/tonsilitis	[1]

ISS4060	*C. diphtheriae *var. *gravis*, non-toxigenic, isolated from patients affected by pharyngitis/tonsilitis	[1]

ISS4746	*C. diphtheriae *var. *gravis*, non-toxigenic, isolated from patients affected by pharyngitis/tonsilitis	[21]

ISS4749	*C. diphtheriae *var. *gravis*, non-toxigenic, isolated from patients affected by pharyngitis/tonsilitis	[21]

NCTC13129	*C. diphtheriae *var. *gravis*, non-toxigenic, isolated from pharyngeal membrane, patient with clinical diphtheria	[2]

***E. coli***		

DH5αMCR	*endA1 supE44 thi-1 *λ^- ^*recA1 gyrA96 relA1 deoR Δ(lacZYA-argF) U196 *ϕ80*ΔlacZ ΔM15mcrA Δ(mmr hsdRMSmcrBC)*	[9]

***Salmonella enterica *serovar Typhimurium (*S*. Typhimurium)**		

NCTC12023	wild type identical to ATCC14028	NCTC, Colindale, UK

**Cell lines**		

Detroit562	human hypopharyngeal carcinoma cells	[20]

### Transformation of competent *C. diphtheriae*

For preparation of electrocompetent cells, 10 ml of an overnight culture of *C. diphtheriae *were inoculated in 200 ml of Brain Heart Infusion (BHI) containing 2% glycine and 15% sucrose, at 37°C in an orbital shaker until an OD_600 nm _of 0.5 was reached. After storing the cells on ice for 15 min, bacteria were harvested by centrifugation (4,000 × g, 4°C), washed thrice with 15% glycerol, and resuspended in 1 ml of 15% glycerol. 100 μl aliquots of the competent cells were frozen in liquid nitrogen and stored at -80°C.

For transformation the aliquots were thawed on ice. Plasmid DNA used for transformation was extracted from *E. coli *strain DH5αMCR, which is unable to methylate DNA. One microgram of plasmid DNA was used to transform *C*. *diphtheriae *cells using a GenePulser II apparatus (Bio-Rad, Munich, Germany) and 200 Ω, 2.5 kV, 25 μF. Electroporated cells were added to 1 ml of HI broth containing 1% glucose, incubated for 2 h at 37°C, and plated on medium containing 10 μg ml^-1 ^kanamycin. Subsequently, transformants were cultivated in the presence of 50 μg ml^-1 ^kanamycin.

### Construction of GFP reporter plasmids and reporter gene assay

For construction of reporter plasmids, promoter regions of the pili- encoding *spaA*, *spaG*, *spaH*, and *spaI *genes were amplified from chromosomal DNA by PCR (for oligonucleotides used see Table 2). The PCR products were inserted into plasmid pEPR1, using the *Nsi*I and *Bam*HI restriction site upstream of the promoterless *gfp*uv gene. Plasmids constructed (Table 3) were used to transform electrocompetent *C. diphtheriae *and resulting plasmid-carrying strains were applied to analyze *spa *promoter activity before and in response to host cell contact. For this purpose, Detroit562 cells were seeded on coverslips 48 h prior to infection. Adhesion assays with Detroit562 cells were carried out as described below (4 μl of the inoculi dried on coverslips were used as no contact control). Subsequently, the infected cells were fixed with 3% *para*-formaldehyde (PFA) in PBS (10 min, room temperature), coverslips were mounted on glass slides using Fluoroprep (Biomerieux, Craponne, France), and imaging was done on a LSM700 confocal laser microscope (Carl Zeiss Micromaging GmbH, Jena, Germany). GFP fluorescence emission of *C. diphtheriae *without and with 1.5 h of host cell contact was determined for ISS4746 and the different reporter plasmids (Table 3) by analyzing between 80 and 140 bacteria for each condition using the open source ImageJ program package (http://rsbweb.nih.gov/ij/index.html).

**Table 2:** Oligonucleotides used in this study

Designation	Sequence (5'→3' direction)	Application
DIP0235-as	ctt ggt tgc cgg agc agc ctc ctt	PCR *spaD*

DIP0235-s	ccg aaa cca aga ccg aga aga ccg tca ag	PCR *spaD*

DIP0237-as	gca cac cag tca gcg cca agt cgc	PCR *spaE*

DIP0237-s	cgc gac tac gga acc gac acg ctg a	PCR *spaE*

DIP0238-as	gaa gtt gaa agg tcg gcc act aca gca a	PCR *spaF*

DIP0238-s	aaa ggg cta cta cat caa cat tcc aga cac	PCR *spaF*

DIP2010asT7	cgc gta ata cga ctc act ata ggg tcc atc acg agg aac gac aac ggt ttt aga	*spaC *probe

DIP2010-s	cgc tac tcc tat ggg caa gca cct act gat att c	*spaC *probe

DIP2011asT7	cgc gta ata cga ctc act ata ggg tgg tgg cga tgg cca gca gtc cga	*spaB *probe

DIP2011-s	tgc agc att cgc cga cga cca acc	*spaB *probe

SondeDIP2013-asT7	ggg ccc taa tac gac tca cta tag gga gga ctg gag tgt tgc gcc g	*spaA *probe

SondeDIP2013-s	ggc gtc gaa aat caa gct gg	*spaA *probe

DIP2223asT7	cgc gta ata cga ctc act ata ggg atc ggt aac ttc ctt acg gaa ctt ctc tgg cag	*spaI *probe

DIP2223-s	ttg ccc gcg gga act atc gac gga	*spaI *probe

SondeDIP2226-asT7	ggg ccc taa tac gac tca cta tag ggc cca gcc cct gcg acg tc	*spaH *probe

SondeDIP2226-s	gga ggg ctg gga ggc agt ca	*spaH *probe

DIP2227asT7	cgc gta ata cga ctc act ata ggg tcg acc ttg gac cag tgg acc tta gcg	*spaG *probe

DIP2227-s	ccg gac aga aga ttg ctg ccg agg ca	*spaG *probe

PromDIP2223-as	cgc ggg atc cag tag ggc gtc ctt tca gga	construction reporter plasmid for *spaI*

PromDIP2223-s	cgc gat gca tgt gac gcc attt tat gta cgc	construction reporter plasmid for *spaI*

PromDIP2226-as	cgc ggg atc cag ggt gtt ttc ctt tca gga	construction reporter plasmid for *spaH*

PromDIP2226-s	cgc gat gca tcg tca aag tta cgg ccg acc	construction reporter plasmid for *spaH*

PromDIP2227-as	cgc ggg atc cag tga aaa cac ctt cta ggg	construction reporter plasmid for *spaG*

PromDIP2227-s	cgc gat gca ttg aac cgg aat cat ttc tta	construction reporter plasmid for *spaG*

PromDIP2013-neu-as	cgc ggg atc ccc ctc aac tta ttt att tgg caa aaa g	construction reporter plasmid for *spaA*

PromDIP2013-neu-s	cgc gat gca tat tgg tga gac tac ttc ctt aaa gct ggt	construction reporter plasmid for *spaA*

**Table 3:** Plasmids used in this study

Plasmid	Description	Reference/Source
pEPR1*_spaA*promoter	promoter sequence (500 bp) of *C. diphtheriae spaA *gene	This study

pEPR1*_spaG*promoter	promoter sequence (500 bp) of *C. diphtheriae spaG *gene	This study

pEPR1*_spaH*promoter	promoter sequence (500 bp) of *C. diphtheriae spaH *gene	This study

pEPR1*_spaI*promoter	promoter sequence (500 bp) of *C. diphtheriae spaI *gene	This study

pEPR1	*gfp*uv, Km^R^, *rep*, *per*, T1, T2	[27]

### RNA preparation, hybridization analyses

Total *C. diphtheriae *RNA was prepared from 20 ml cultures using the NucleoSpin RNA II Kit (Macherey Nagel, Düren, Germany). For the generation of antisense probes, internal DNA fragments of the corresponding genes were amplified by PCR (oligonucleotide primers for the different probes are listed in Table 2). The reverse primers encoded the promoter region for T7 polymerase, which allowed *in vitro *transcription of probes using T7 polymerase and subsequent labelling with DIG RNA-labeling mix (Roche, Mannheim, Germany). RNA (2.5 μg per time point) was spotted onto nylon membranes using a Schleicher & Schuell (Dassel, Germany) Minifold I Dot Blotter. Hybridization of digoxigenin-labelled RNA probes was detected with X-ray films (Hyperfilm MP; GE Healthcare, Munich, Germany) using alkaline phosphatase-conjugated anti-digoxigenin Fab fragments and CSPD as light-emitting substrate as recommended by the supplier (Roche, Mannheim, Germany).

### SDS-PAGE and Western blotting

Cell extracts of *C. diphtheriae *strains were separated by Tricine-buffered 9.5% SDS gels as described [26], proteins were transferred onto a polyvinylidene difluoride membrane (PVDF, Immobilon-P, pore size 0.45 μm, Millipore, Bedford, MA, USA) by semi-dry electroblotting, and immunodetection of SpaD was performed with antibodies directed against this pili subunit produced in rabbits [13]. Antibody binding was visualised by using appropriate anti-antibodies coupled to alkaline phosphatase (Sigma-Aldrich, Taufkirchen, Germany) and the BCIP/NBT alkaline phosphatase substrate (Sigma-Aldrich, Taufkirchen, Germany).

### Atomic force microscopy (AFM)

Overnight cultures grown in 20 ml HI broth were washed five times in 20 ml ice cold distilled water and finally resuspended in 10 ml ice cold distilled water (centrifugation steps: 10 min 4,500 × g; resuspension by vortexing). 5 μl of each sample were fixed on a glass slide by drying using compressed air. An AFM instrument (MFP-3D, Asylum Research, Santa Barbara, CA) with standard silicon cantilever probes (NCH-W, Nanosensors, Neuchatel, Switzerland) was used under ambient laboratory conditions and operated in tapping mode [26].

### Measurement of transepithelial resistance

D562 cells were seeded in transwells (6.5 mm, 0.4 μm, polyester membrane, 24 well plate, Corning Costar) at a density of 5 × 10^4 ^cells per well and cultivated in DMEM (Dulbecco's modified Eagle's medium, PAA; high glucose, 10% FCS, 2 mM glutamine) for 14 days until they build a transepithelial resistance of at least 1600 Ω·cm^-2^. Bacteria were subcultured (OD_600 _of 0.1 from overnight cultures) in 20 ml HI broth for 3.5 h. The pellet was resuspended in 500 μl 1 × PBS. 50 μl of the suspension were used for infection. Measurements of transepithelial resistance of D562 cells during the infection with *C. diphtheriae *were carried out with a volt-ohm-meter (EVOM2, World Precision Instruments, Berlin, Germany) every 30 min. After 3 h the supernatant of infected D562 cells was removed and the cells were incubated in fresh DMEM overnight to avoid detrimental effects of excessive bacterial growth.

### Adhesion assays

D562 cells were seeded in 24 well plates (bio-one Cellstar, Greiner, Frickenhausen, Germany) at a density of 2 × 10^5 ^cells per well 48 h prior to infection. Bacteria were subcultured (OD_600 _of 0.1 from overnight cultures) in HI broth for 3.5 h and adjusted to an OD_600 _of 0.2. A master mix of the inoculum was prepared in DMEM without penicillin/streptomycin at a MOI of 200 (viable counts experiments). The plates were centrifuged for 5 min at 500 × g to synchronize infection and subsequently incubated for 1.5 h. The cells were washed with PBS nine times, detached with 500 μl trypsin solution (0.12% trypsin, 0.01% EDTA in PBS) per well (5 min, 37°C, 5% CO_2_, 90% humidity) and lysed with 0.025% Tween 20 for 5 min at 37°C. Serial dilutions were made in pre-chilled 1 × PBS and plated on blood agar plates to determine the number of colony forming units (cfu). From this, the percentage of invasive bacteria was calculated [24].

### Epithelial cell invasion model

D562 cells were seeded in 24 well plates (bio-one Cellstar, Greiner, Frickenhausen, Germany) at a density of 2 × 10^5 ^cells per well 48 h prior to infection. Overnight cultures of *C. diphtheriae *grown in HI were re-inoculated to an OD_600 _of 0.1 in fresh medium and grown aerobically for another 3.5 h. An inoculum of approximately 1.6 × 10^8 ^bacteria ml^-1 ^(MOI = 200) was prepared in DMEM without penicillin/streptomycin and 500 μl per well were used to infect the D562 cells. The plates were centrifuged for 5 min at 500 × g to synchronize infection and subsequently incubated for 1.5 h (37°C, 5% CO_2_, 90% humidity). The cells were washed thrice with PBS and subsequently 500 μl of DMEM containing 100 μg ml^-1 ^gentamicin were applied to each well to kill remaining extracellular bacteria. After 2, 8.5 and 18.5 h of incubation the cell layers were washed thrice with PBS, detached by adding 500 μl trypsin solution (0.12% trypsin, 0.01% EDTA in PBS) per well (5 min, 37°C, 5% CO_2_, 90% humidity) and lysed for 5 min at 37°C with 0.025% Tween 20 to liberate the intracellular bacteria. Serial dilutions of the inoculum and the lysates were plated on blood agar plates to determine the number of colony forming units (cfu).

### Immuno-fluorescence

For immuno-fluorescence staining an antibody against the *C. diphtheriae *surface proteome was used, which was raised in rabbits. For antibody generation, surface proteins were prepared as described [24]. As secondary antibodies Alexa-Fluor 488 (green) goat anti-rabbit IgGs and Alexa-Fluor 568 (red) goat anti-rabbit IgGs were used. Phalloidin Alexa-Fluor 647 was used for staining the cytoskeleton of D562 cells. All antibodies were diluted in blocking solution (2% goat serum, 2% BSA in PBS). Cell lines were seeded on round coverslips in 24 well plates 48 h prior to infection and fixed after the respective assay with 3% PFA in PBS (10 min at room temperature). For immuno-fluorescence staining the preparations were washed thrice with 1 × PBS and incubated with primary antibodies for at least 1 h at room temperature, washed thrice with PBS again, and subsequently incubated with Alexa-Fluor 488 (green) goat anti-rabbit for 45 min. After permeabilization with 0.1% Triton X-100 (5 min room temperature) and three washing steps with PBS, staining with Alexa-Fluor 568 (red) goat anti-rabbit was carried out as described above. F-actin was stained in parallel with Phalloidin-Alexa-Fluor 647 (blue). Coverslips were mounted on glass slides using Fluoroprep (Biomerieux, Craponne, France). Imaging was done on an AxioVert 200 M inverted optical microscope (Carl Zeiss Micromaging GmbH, Jena, Germany).

## Authors' contributions

LO carried out invasion assays, fluorescence microscopy, TER measurements, and RNA and protein analyses, MHö carried out adhesion and invasion analysis, AFM experiments were carried out in cooperation with JR and TES, MHe supported LO and MHö in respect to cell culture, AB supervised the experiments of LO and MHö and was responsible for the draft and final version of the manuscript. All authors read and approved the final manuscript.
